# Transformers for autonomous recognition of psychiatric dysfunction via raw and imbalanced EEG signals

**DOI:** 10.1186/s40708-023-00201-y

**Published:** 2023-09-09

**Authors:** Neha Gour, Taimur Hassan, Muhammad Owais, Iyyakutti Iyappan Ganapathi, Pritee Khanna, Mohamed L. Seghier, Naoufel Werghi

**Affiliations:** 1 Khalifa University Center for Autonomous Robotic System and Cyber-Physical Security System Center, Department of Electrical Engineering and Computer Science, Khalifa University, Abu Dhabi, United Arab Emirates; 2 Departement of Electrical and Computer Engineering, Abu Dhabi University, Abu Dhabi, United Arab Emirates; 3 Department of Computer Science and Engineering, Indian Institute of Information Technology, Design and Manufacturing, Jabalpur, India; 4 Healthcare Engineering Innovation Center, Department of Biomedical Engineering, Khalifa University, Abu Dhabi, United Arab Emirates

**Keywords:** EEG Classification, Transformer Networks, Multivariate Time-series Classification, Class Imbalance, Psychiatric Dysfunction

## Abstract

Early identification of mental disorders, based on subjective interviews, is extremely challenging in the clinical setting. There is a growing interest in developing automated screening tools for potential mental health problems based on biological markers. Here, we demonstrate the feasibility of an AI-powered diagnosis of different mental disorders using EEG data. Specifically, this work aims to classify different mental disorders in the following ecological context accurately: (1) using raw EEG data, (2) collected during rest, (3) during both eye open, and eye closed conditions, (4) at short 2-min duration, (5) on participants with different psychiatric conditions, (6) with some overlapping symptoms, and (7) with strongly imbalanced classes. To tackle this challenge, we designed and optimized a transformer-based architecture, where class imbalance is addressed through focal loss and class weight balancing. Using the recently released TDBRAIN dataset (n= 1274 participants), our method classifies each participant as either a neurotypical or suffering from major depressive disorder (MDD), attention deficit hyperactivity disorder (ADHD), subjective memory complaints (SMC), or obsessive–compulsive disorder (OCD). We evaluate the performance of the proposed architecture on both the window-level and the patient-level. The classification of the 2-min raw EEG data into five classes achieved a window-level accuracy of 63.2% and 65.8% for open and closed eye conditions, respectively. When the classification is limited to three main classes (MDD, ADHD, SMC), window level accuracy improved to 75.1% and 69.9% for eye open and eye closed conditions, respectively. Our work paves the way for developing novel AI-based methods for accurately diagnosing mental disorders using raw resting-state EEG data.

## Introduction

Electroencephalography (EEG) signals are widely used in many applications related to brain–computer interfacing [1, 2], motor imagery classification [3–5], emotion recognition [6, 7], neuroscience [8, 9], and biomedical engineering [10, 11]. In the field of neuroscience, EEG signals can serve as useful biomarkers and clinically relevant features for the identification of neurological and mental dysfunctions. These features are further used to monitor and improve the treatment plan for patients. Automated classification of EEG signals has numerous applications in the area of neurological and mental disease diagnosis and management.

EEG has various advantages, making it one of the most widely used tool in the clinical setting, like non-invasive acquisition, portability, cost-effective, and offering high temporal resolution. However, multiple challenges are associated with EEG signal processing and automated neurological dysfunction classification tasks. The EEG signals have a low signal-to-noise ratio, low spatial resolution, and high inter-subject variability [12]. Although the high temporal resolution is beneficial as rapid changes in brain activity can be observed [13], handling such data with limited computation capabilities can be challenging. Raw EEG signals are also associated with various artifacts introduced by different sources that interfere with feature extraction and classification. Typically, raw EEG signals are pre-processed to remove these artifacts [14, 15], including pulse and respiratory artifacts, eye movements, muscle activity, and other movement artifacts. In some cases, the EEG pre-processing techniques might result in the loss of important information. An automated model for mental dysfunction classification using raw EEG signals is highly desirable as it might open the possibility for more ecological applications of EEG for large-scale screening procedures. In addition to the challenging nature of raw EEG data, the classification of different mental disorders is notoriously difficult because many of these disorders share similar symptoms, and many of them can be defined as spectrum disorders or syndromes with no clear cutoffs [16]

With the recent advances in deep learning, methods are proposed for diverse applications like computer vision, natural language processing (NLP), time series, and biomedical applications. The deep learning methods have produced state-of-the-art performance exceeding human experts in some cases. Deep learning methods need copious amounts of data and a balanced sample distribution. Implementing deep learning methods for biomedical applications is still a challenging task due to data scarcity and prevalent class imbalance. Recently, Transformer models have shown tremendous success in NLP and multivariate analysis. They are praised for their capacity to model self and mutual attention between serial data [17, 18]. EEG signal is multivariate time series data having information on multiple electrodes for a fixed amount of time. It reflects the activation of multiple brain networks that interact at different spatio-temporal scales. We hypothesize that such multivariate EEG signals can be best captured and interpreted using a transformer model.

The proposed work aims to implement a transformer model for classifying mental dysfunctions using raw EEG data. To effectively evaluate the performance of the transformer model, we have used the publicly available TDBRAIN dataset [19].

The contributions of this work are summarized as follows:

- Transformer model is implemented for challenging raw EEG data of psychiatric patients without pre-processing and feature extraction.
- Comparative analysis of multi-class neurological dysfunction classification model on eye-open and eye-closed resting state raw EEG data.
- Analysis of various methods to curb the class imbalance issue in the publicly available TDBRAIN dataset.
- The performance is analysed on patient-level in addition to window-level decisions, which are generally used in EEG classification frameworks.

The work is organized as follows. Section 2 discusses state-of-the-art techniques for classifying EEG signals. Section 3 describes the proposed transformer architecture methodology for classifying mental dysfunction. The dataset used for experimental evaluation of the performance of the proposed algorithm is also discussed here. The evaluation criteria and results are discussed in Sect. 4. Finally, we conclude with a summary of main findings and some important questions that warrant future research in Sect. 5.

## Related work

The EEG signal processing majorly comprises traditional machine learning approaches [20] and deep learning methods [12]. The conventional machine learning methods include pre-processing, relevant feature extraction, and classification using machine learning classifiers. The machine learning methods differ in how EEG data is treated before the feature extraction step in the time and frequency domain. Initial techniques in the literature include handcrafted feature extraction from five major frequency bands: alpha, beta, theta, delta, and gamma. Alhudhaif [21] implemented an approach to extract 25-time domain features from raw, alpha ($8-13$ Hz), beta ($13-30$ Hz), theta ($4-8$ Hz), and delta ($0.5-4$ Hz) frequency bands. The final 125 features are classified using One-Against-All (OVA) approach. The adaptive synthetic (ADASYN) sampling method is combined with OVA for multi-class imbalanced EEG signals classification. Hosseinifard et al. [22] proposed a similar approach for binary classification of EEG signals into depressive disorders and healthy categories. The bandpass Butterworth filter is applied to raw EEG signals to extract delta, theta, alpha, and beta bands. Correlation dimension, Higuchi, DFA, and Lyapunov exponent methods were further applied on frequency bands to extract features for all 19 EEG channels. Linear discriminate analysis (LDA), Logistic regression (LR), and *k*-nearest neighbor (KNN) classifiers are used for the binary classification. Das et al. [23] proposed machine learning and deep learning approaches on the EEG signal’s discrete wavelet transform (DWT) scalograms using SVM, RF, AdaBoost, and CNN classifiers. Bajaj et al. [24] utilized time–frequency representation (TFR) of EEG signal using smoothed pseudo-Wigner–Ville distribution (SPWVD) for seizure classification. Handcrafted features like angular second moment (ASM), contrast (CON), mean-to-standard deviation ratio (MSR), and area are extracted and applied to the least square SVM for classification.

Emre et al. [25] implemented a machine learning-based approach for multi-class classification of EEG signals. Pre-processing and artifact correction is done on the EEG dataset, and features are extracted from four frequency bands. An unbalanced dataset for nine classes of psychiatric disorders is used for the experiments using classifiers, namely, C5.0, random forest (RF), support vector machines (SVM), and artificial neural networks (ANN). Under-sampling and oversampling methods are implemented on the dataset for comparison. The handcrafted feature extraction methods rely on expert knowledge related to EEG data which may not be as robust for classification.

Deep learning-based methods have recently exhibited superior performance compared to traditional machine learning approaches. Deep learning methods eliminate the need for feature engineering and rely on deep learning networks for automatic feature learning. Convolutional neural networks (CNN), deep belief networks (DBN), recurrent neural networks (RNN), stacked auto-encoders (SAE), and transformers are some of the commonly used architectures in literature for the classification of EEG signals. Although neurological dysfunction classification is a well-established area of research, most machine learning and deep learning-based methods focus mainly on binary classification of diseases like ADHD, SMC, OCD, MDD, post-traumatic stress disorder (PTSD), schizophrenia, etc. [26]. In addition, most of the works used pre-processed EEG data instead of focusing on raw EEG data. Some of the recently published deep learning methods for EEG classification are discussed further. Moghaddari et al. [27] proposed a CNN architecture for diagnosing ADHD in children using continuous mental task EEG. The data is pre-processed, and frequency bands are separated to construct the CNN network’s input images. Another frequency bands-based approach is proposed by Uyulan et al. [28] for classifying EEG signals into healthy and MDD classes. ResNet-50, MobileNet, and Inception-v3 CNN models are applied to topographic maps of frequency bands EEG signals.

For the raw EEG sinal classification few methods are also proposed in the literature. Supakar et al. [29] proposed an RNN–LSTM based approach for Schizophrenia classification from EEG data. The dimension of the EEG data is reduced using principal component analysis (PCA) and treated as a multi-variate time series signal for the LSTM architecture. Erguzel et al. [30] proposed a traditional machine learning approach using SVM, KNN, ANN, and Naive Bayes methods for trichotillomania and OCD classification. Based on the literature review, it can be concluded that the multi-class classification for mental dysfunctions is less explored. Lawhern et al. [31] proposed a compact convolutional neural network for EEG-based BCIs. The raw EEG signals from four publicly available datasets were epoched, and the resulting EEG segments were used as inputs for the CNN-based architecture. Another similar approach was proposed by Schirrmeister et al. [32], named DeepConvNet, for raw EEG data. The architecture comprised four blocks of convolutional and max-pooling operations followed by a dense softmax classification layer. Moreover, transformer-based architectures have also been proposed in the literature to classify EEG signals. For instance, Xie et al. [33] proposed a transformer-based approach combining deep learning with spatial–temporal information from raw EEG data for motor imagery classification, in addition to five different architectures integrating transformer and CNN architectures. Song et al. [34] recently proposed a compact convolutional transformer for EEG-based motor image classification and emotion recognition tasks, tested on for three publicly available datasets. To handle raw EEG data, the suggested architecture comprised a convolutional module, a self-attention module, and a fully connected classifier.

From the literature review, it can be concluded that EEG signals classification is widely used for motor imagery identification and epilepsy detection. Various methods were also proposed for classifying different neurological disorders. However, multi-class EEG-based classification of psychiatric dysfunction is scarce. Perhaps, most importantly, it can be noted that most deep learning methods use EEG signals as input in the frequency domain rather than raw EEG signals in the time domain. The motivation of this work is to fill the gap in current literature by proposing a deep learning based method using raw EEG signals in the time domain for multi-class psychiatric dysfunction classification. The strength of our proposed approach lies in using raw EEG signals in the time domain without any subsequent transformation to frequency bands or image-based features. Although transformer models were previously implemented for EEG-based classification, our approach is among the few available studies that addressed the challenging question of classification of mental dysfunction. As far as we know, our work is the first to propose a transformer model for raw EEG signals using the TDBRAIN dataset, while addressing the problem of class imbalance.

## Materials and methods

TDBRAIN dataset for psychiatric dysfunction classification is used for experimentation. A transformer-based method is used for the classification of raw EEG signals into five categories of neurological dysfunction is discussed here.

### Data set used for proposed work

The proposed method is employed on the EEG signals from the recently released ”two decades brain clinics research archive for insights in neurophysiology (TDBRAIN) dataset” [19]. The dataset comprises 1274 raw EEG data of patients with a clinical lifespan of $5-89$ years. The EEG signals are captured while giving specific instructions to patients and following standard protocols. The psychophysiological recordings are captured using a 10–10 electrode international system at a sampling rate of 500 Hz.

**Table 1:** TDBRAIN dataset for resting state raw EEG data with eye open and eye close

S.No.	Indication	No. of EEG sessions	Formal Dx
1.	MDD	426	198
2.	ADHD	271	141
3.	SMC	119	-
4.	OCD	75	58
5.	Tinnitus	33	-
6.	Insomnia	32	32
7.	Parkinson	27	17
8.	Burnout	10	10
9.	Dyslexia	26	20
10.	Chronic pain	14	14
11.	Others	80	-
12.	Unknown	255	-
13.	Healthy	47	-

The final raw EEG signals include 26-channel recordings. The EEG recordings were taken while the patient was resting with eyes open (EO) and eyes closed (EC) for 2 min each. During the EO task, the patients were asked to focus on a red dot at the center of a computer screen while in resting state. The EC samples were recorded in the patient with closed eyes and retaining the same position as the EO task. Differences between EO and EC have been previously reported in terms of topography as well as power levels [35]. Among 1274 patients, EEG data was captured for mental dysfunctions, namely, major depressive disorder (MDD), attention deficit hyperactivity disorder (ADHD), subjective memory complaints (SMC), obsessive–compulsive disorder (OCD), tinnitus, Insomnia, Parkinson, Burnout, Dyslexia, Chronic pain, and Healthy. Individual distribution of each class is shown in Table 1.

### Pre-processing and data preparation

The proposed work focuses on classifying five main classes highlighted by Dijk et al. [19]: ADHD, MDD, OCD, SMC, and healthy. As shown in Table 1, ADHD, MDD, and SMC classes have a relatively higher number of samples than OCD and healthy classes. Based on this observation, experiments were performed on different cost functions to address the class imbalance issue. In addition to five class classifications, experiments with three majority classes were also performed. The TDBRAIN dataset comprises EO and EC EEG signals for each patient. The proposed model is implemented for both types of signals separately. Bandpass filtering is applied to the raw EEG signal with 50 Hz frequency [35]. Each EEG signal is then down-sampled using a sampling frequency of 100 Hz to reduce the temporal resolution of the EEG time series data. The EEG data is finally cropped by 3s from both ends to remove any potential artifacts introduced due to the filtering and down-sampling process. Z-score normalization is applied to the EEG signal as follows:

1 $$S^{*} = \frac{S-\mu }{\sigma }$$

where *S* is the original EEG signal and $S^{*}$ is the normalized EEG signal. $\mu$ is the mean value of the signal along the electrodes, and $\sigma$ is the standard deviation. The pre-processed EEG signals are divided into epochs of window size 10 seconds with 2 seconds overlapping. Each window comprises all 26 electrodes of an EEG signal.

**Fig. 1:**
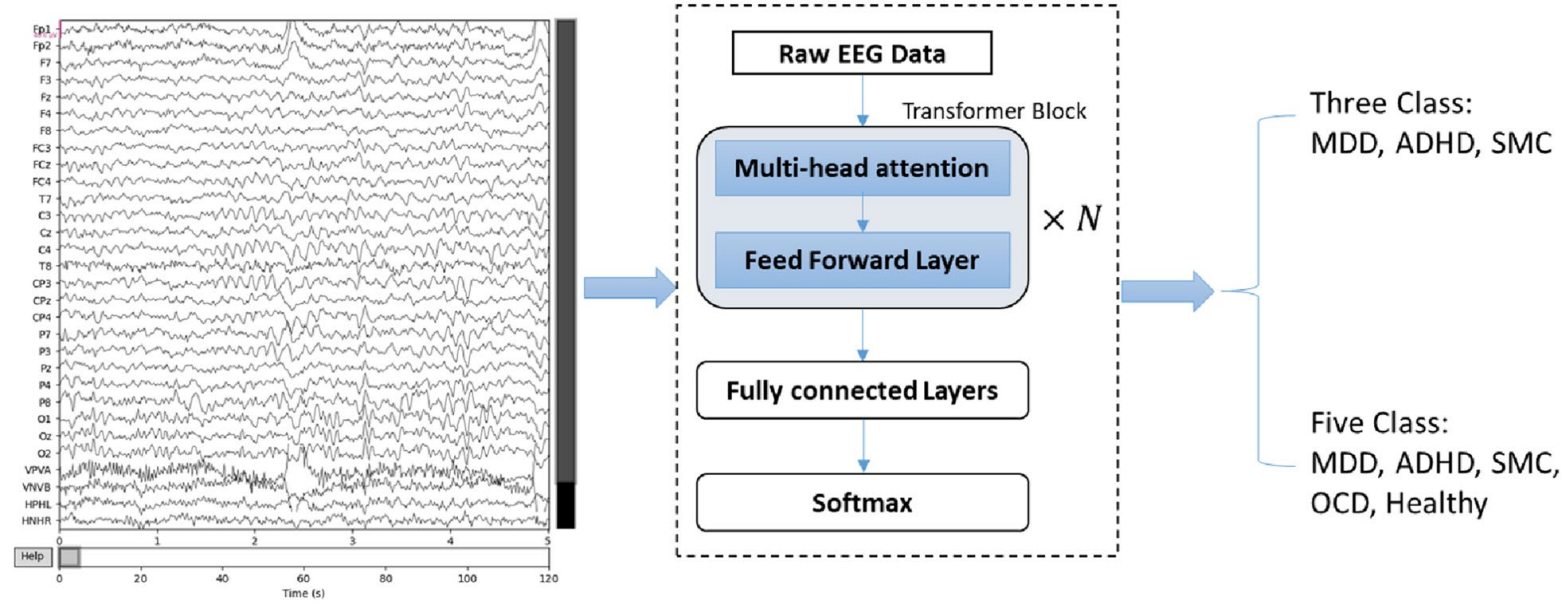
Proposed framework for neurological dysfunction classification using transformer-based model on raw EEG data

### Model architecture

**Fig. 2:**
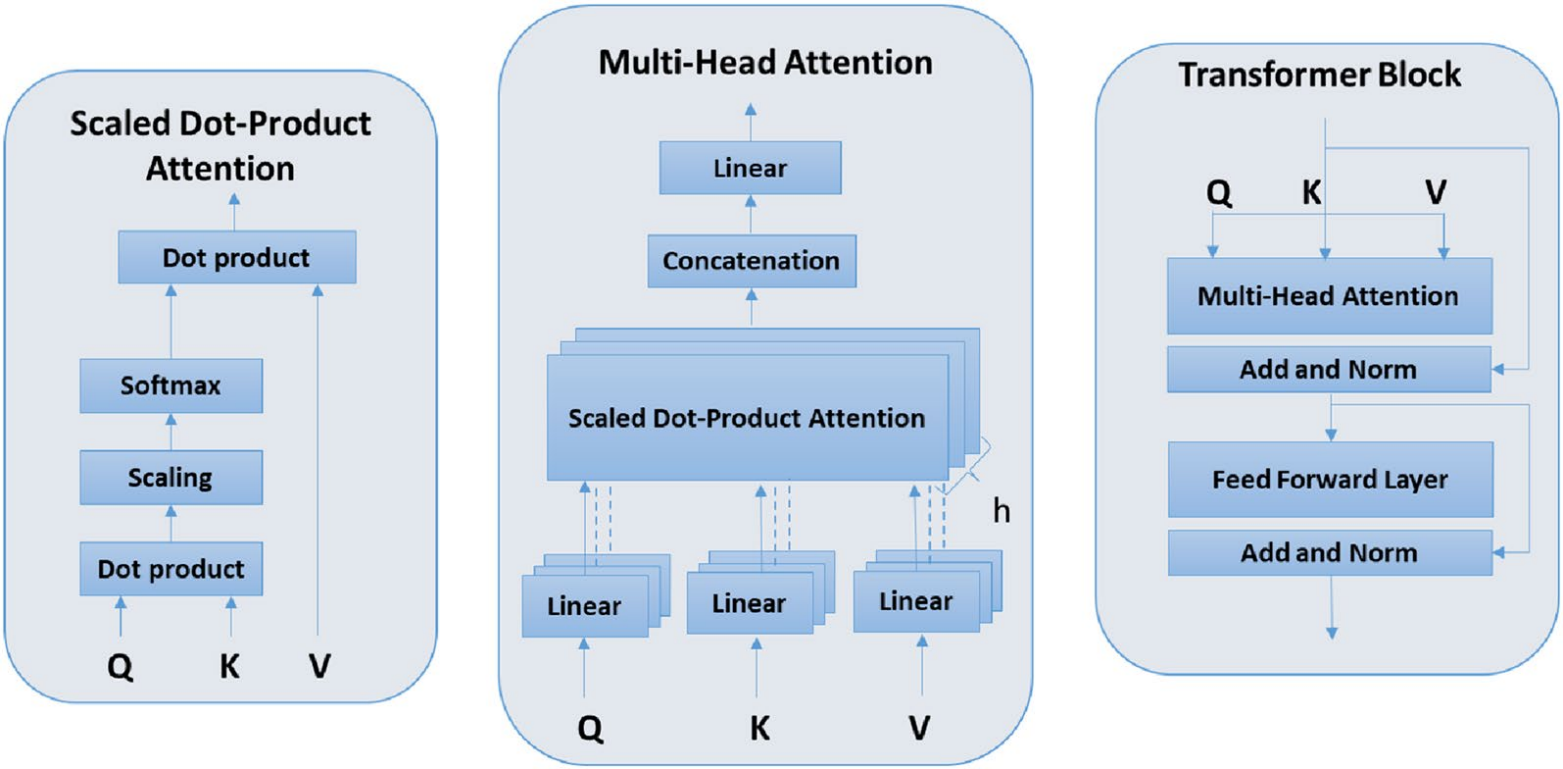
Transformer Modules (Left to Right): Scaled dot-product attention, Multi-head attention, and Transformer module

Transformer network architecture originally proposed by Vaswani et al. [17] is adopted for classifying EEG signals from the TDBRAIN dataset as shown in Fig. 1. The raw EEG signal is pre-processed as described in Sect. 3.2 and applied to the transformer network for three and five-class classification. The transformer network follows an encoder–decoder structure. In this work, the encoder part of the transformer is used for feature extraction, followed by the softmax layer for multi-class classification. The transformer encoder comprises two sub-layers: a multi-head attention layer and a fully connected feed-forward layer. A residual connection is around these sub-layers, followed by layer normalization operations. The multi-head attention layer comprises of scalar dot-product attention block as shown in Fig. 2. The input vector is multiplied with three different weight matrices to construct three vectors. The query vector (Q), key vector (K), and values vector (V) are applied in the scaled dot-product attention for weighted value calculation, as shown in the following equation:

2 $$Attention(Q,K,V) = softmax\left( {\frac{{QK^{T} }}{{\sqrt {d_{k} } }}} \right)V$$

The combination of several such scaled dot-product attention layers in parallel is applied to create a multi-head attention layer. The multiple attention layers allow the model to focus on features from different subspaces at different locations. The output of the multi-head attention layer is calculated as follows:

3 $$\begin{aligned} MultiHead(Q,K,V) = Concat(Att_1,...., Att_h) W^O \end{aligned}$$

where

4 $$\begin{aligned} Att_i = Attention_i(Q,K,V) \end{aligned}$$

The multi-head attention layer is combined with a fully connected feed-forward layer to create a transformer block. A typical transformer architecture for classification comprises multiple transformer blocks. We have used four encoder transformer blocks, each having eight attention heads for the multi-attention layer.

Recently, the transformer network originally proposed for natural language processing is adapted for multi-variate time series data [36]. The EEG signal as input to the transformer network is formulated as multi-variate time series data with electrodes as variables. In this work, each training sample is represented as $X \in \textbf{R}^{m \times e} = [x_1,x_2,...,x_m ]$. The training sample constitutes *m* feature vectors as input of length as number of electrodes (*e*).

### Class imbalance

The proposed method is implemented on the TDBRAIN dataset for neurological dysfunction classification. In the original study published by Dijk et al. [19], more emphasis was given to the five main classes of mental dysfunction: MDD, ADHD, OCD, SMC, and Healthy. We selected these classes to evaluate the transformer model in our work for the first section of the experimental setup. The distribution of these classes (as shown in Table 1) shows the prevalence of class imbalance in the TDBRAIN dataset. The number of samples of MDD, ADHD, and SMC classes is relatively higher than in OCD and healthy classes. The second experimental section involves implementing transformer models for three majority classes, MDD, ADHD, and SMC. An ablation study is also performed using three different loss functions to address the class imbalance issue in the TDBRAIN dataset. The transformer model is trained with categorical cross-entropy (CCE), weighted sparse categorical cross-entropy (WCCE), and focal loss functions. The CCE loss adjusts model weights during training [37]. The CCE loss function is calculated between the ground truth label and the predicted probability score of each class and defined as follows:

5 $$\begin{aligned} L_{CCEq} = - \sum _{i=1}^{n} t_i \log (p_i) \end{aligned}$$

where *n* is the number of classes, $t_i$ is ground truth label, $p_i$ is probability by softmax function for $i^{th}$ class. The WCCE loss function is implemented by applying the class weights of each class from the training dataset. The class weights for each class are calculated by the method given by King et al. [38]:

6 $$\begin{aligned} W_i = \frac{N}{k \times N_i} \end{aligned}$$

where $W_i$ is the weight of class *i*, *N* is the total number of EEG signals, *k* is the total number of classes, and $N_i$ is the number of EEG signal samples in class *i*. The class weights are applied during transformer weight learning and penalize the classification of minority class into majority class. The WCCE loss function is defined as follows:

7 $$\begin{aligned} L_{WCCE} = - \sum _{i=1}^{n} W_i t_i \log (p_i) \end{aligned}$$

Training the deep learning models using focal loss [39] is another method for the dataset with class imbalance. The focal loss function down weights the influence of majority classes, resulting in efficient learning for minority classes. The focal loss is calculated similarly to the vanilla CCE loss function with an added modulating factor for class imbalance. The focal loss is defined as follows:

8 $$\begin{aligned} L_{FL} = - \sum _{i=1}^{n} (i-p_i)^\gamma \log (p_i) \end{aligned}$$

where $\gamma$ is a modulating factor that can be tuned according to the dataset. In our work, the values of $\gamma$ are selected based on the through empirical study performed for object detection in class imbalance conditions. The work by Lin et al. [39] suggest the best value for $\gamma$ as 2. In addition a grid analysis is done on the proposed model to get the best value for $\gamma$ as 0.5 for TDBRAIN dataset in some cases (as shown in subsequent Sect. 4).

### Training setup

The EEG classification in literature is divided into within-individual and cross-individual training setups. These setups differ in how training and testing sets are extracted from the dataset. In a within-individual setup, multiple sessions of the same individual are divided into training and testing sets. Within-individual setup leads to higher accuracy during testing. However, in the real-life scenario, the cross-individual setup is more relevant. The model trained on training individuals is tested on new individuals. The cross-individual setup provides more robust and generalized models due to information transfer among individuals. This work uses a cross-individual setup to create training, validation, and testing sets from the TDBRAIN dataset. The five and three class samples are divided into $80\%$, $10\%$, and $10\%$ for training, validation, and testing sets, respectively.

The transformer model is trained and tested with window-level signals as described in Sect. 3.2. During the testing stage, the window-level predictions are aggregated using a majority voting technique to obtain a patient-level prediction. The classification scores for each window for a particular patient are aggregated together. Majority voting is applied to the windows to get the final decision for each patient.

The architecture hyper-parameters were chosen according to the original transformer encoder module [17]. Based on empirical experimentation, the hyper-parameters of the transformer model were subsequently tuned during model validation on the raw EEG signals of the TDBRAIN dataset. Different values of attention heads, head size, number of transformer blocks, and dense layer size were explored during the experimentation. Based on the performance, the values for attention heads, head size, number of transformer blocks, and dense layer were fixed at 4, 32, 4, and 512, respectively. The multi-head attention layer was implemented with a ReLU activation function with a dropout rate of 0.25. The training hyper-parameter tuning was done for learning rate, batch size, and number of epochs. The transformer model was trained using the Adam optimizer with a 0.001, 0.0001, and 0.0005 learning rate. The best result was obtained with a learning rate of 0.0005. The models were trained on NVIDIA Tesla P100 GPU with a memory size of 15GB, for which the batch size was set to 4 to accommodate the computational capabilities. During initial tests, we explored training for 100, 200, and 300 epochs for hyper-parameter tuning of the number of epochs. The number of epochs was set to 100 based on the achieved performance during these initial tests. The model was trained for 100 epochs with the early-stopping and checkpoint method to choose the best model among the trained models. The best models were selected based on the highest validation accuracy, and the training was stopped using a patience parameter of 20. The code is implemented on Python 3.8 on the TensorFlow backend with CUDA: 9.1.85 and cuDNN: 7.1.1 versions.

## Results and discussion

The proposed method is applied to raw EEG signals of the TDBRAIN dataset. The models are tested on holdout 10% of the TDBRAIN dataset. The performance is evaluated and compared on the confusion matrix-based parameters. Accuracy, F1-score, Precision, and Recall parameters are calculated and compared with different experiments. The F1-score (12) is defined as the harmonic mean of precision and recall parameters. Accuracy (9), Precision (10), and recall (11) are defined in terms of true positive (*TP*), true negative (*FP*), false positive (*FP*), and false negative (*FN*) as follows:

9 $$\begin{aligned} Accuracy= {} \frac{TP+TN}{TP+TN+FP+FN} \end{aligned}$$

10 $$\begin{aligned} Precision= {} \frac{TP}{FP+TP} \end{aligned}$$

11 $$\begin{aligned} Recall= {} \frac{TP}{TP+FN} \end{aligned}$$

12 $$\begin{aligned} F1-Score= {} 2 \times \left[ \frac{Precision \times Recall}{Precision + Recall} \right] \end{aligned}$$

We have compared the results of the proposed method for the EO and EC EEG signals based on accuracy, F1-score, precision, and recall parameters. The final decision is made window level and patient level for the three- and five-class classification. It is observed that the performance with patient-level decisions is relatively better than window-level decisions. Tables 2 and 3 summarize the performance of the transformer model for five-class classification on EO and EC samples. The proposed method shows superior performance using EO samples in terms of accuracy for all loss functions. However, better performance is observed in terms of F1-score, precision, and recall five-class classification performance with EC samples. The transformer model with WCCE loss function shows improved performance with respect to CCE loss function in the case of EO five-class classification for window level decision. The best accuracy of $68.49\%$ is achieved with focal loss ($\gamma$ = 2) in patient-level decision for EO experiment and $68.49 \%$ for EC experiment with focal loss ($\gamma$ = 0.5) in patient-level decision.

**Table 2:** Performance of transformer model for EEG signals with eyes open on different methods for five class (ADHD, MDD, OCD, SMC, and Healthy) classification

Decision	Method	Accuracy	F1-score	Precision	Recall
Window-level	Trans + CCE	**63.21**	41.99	42.51	41.49
	Trans + Focal Loss ($\gamma = 2)$	61.25	40.91	41.65	40.20
	Trans + Focal Loss ($\gamma = 0.5)$	60.27	37.59	36.38	41.27
	Trans + WCCE	53.30	**43.67**	**44.64**	**42.73**
Patient-level	Trans + CCE	64.38	42.90	42.81	43.96
	Trans + Focal Loss ($\gamma = 2)$	**68.49**	43.29	45.00	41.72
	Trans + Focal Loss ($\gamma = 0.5)$	61.64	38.78	39.66	40.90
	Trans + WCCE	57.53	**45.03**	**45.33**	**44.75**

The best performance is in bold

**Table 3:** Performance of transformer model for EEG signals with eyes close on different methods for five class (ADHD, MDD, OCD, SMC, and Healthy) classification

Decision	Method	Accuracy	F1-score	Precision	Recall
Window-level	Trans + CCE	61.74	**46.57**	51.24	**47.83**
	Trans + Focal Loss ($\gamma$ = 2)	55.28	31.34	35.77	32.70
	Trans + Focal Loss ($\gamma$ = 0.5)	**65.85**	45.60	**51.99**	45.03
	Trans + WCCE	49.61	36.94	37.06	37.72
Patient-level	Trans + CCE	67.12	**49.84**	**63.55**	**49.54**
	Trans + Focal Loss ($\gamma$ = 2)	57.53	32.00	40.79	32.84
	Trans + Focal Loss ($\gamma$ = 0.5)	**68.49**	44.59	49.51	45.11
	Trans + WCCE	52.05	36.89	35.79	38.25

The best performance is in bold

**Fig. 3:**
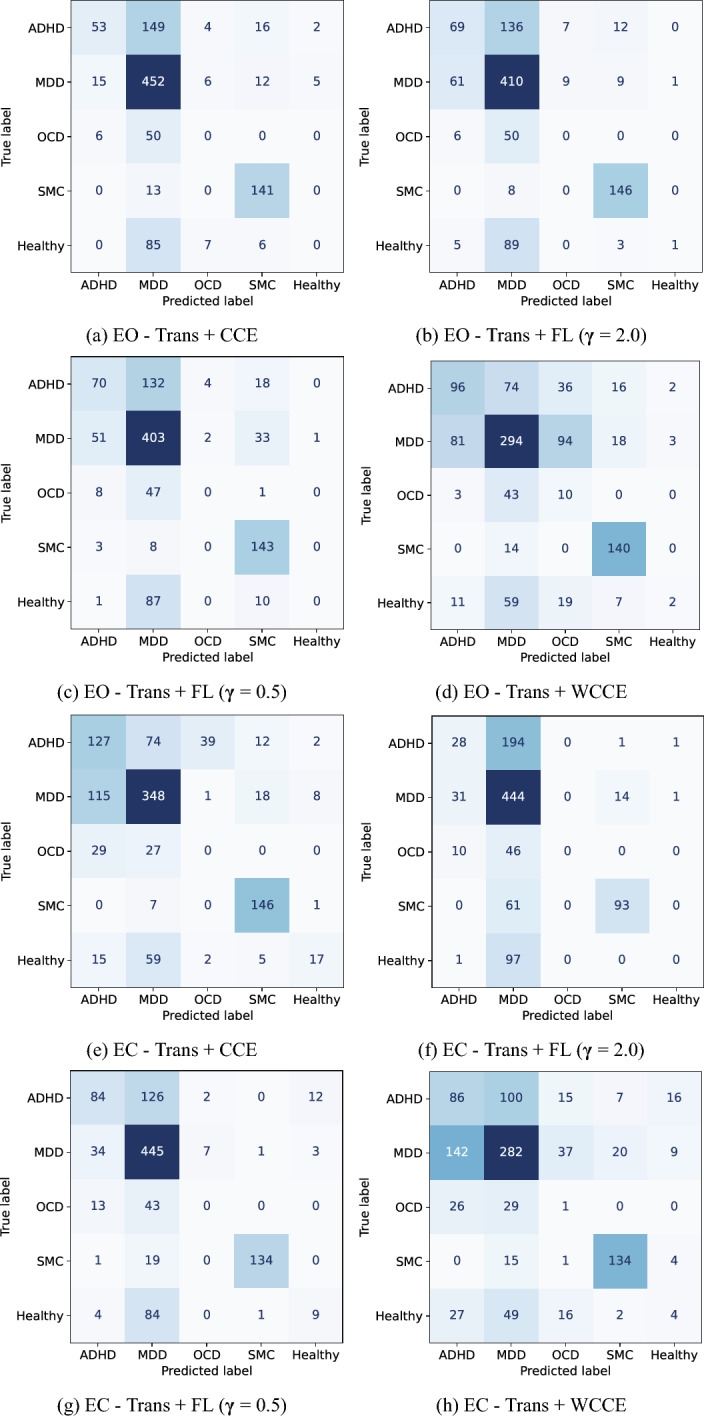
Confusion matrices for the five-class classification eye open and eye closed samples

The confusion matrix of window level decision for each experiment shows the advantage of using WCCE and focal loss function for classification (as shown in Fig. 3). The model trained on EO samples is biased toward the ADHD class, and the minority classes, i.e., OCD and healthy, are not recognized (Fig. 3(a)). However, it can be seen in Fig. 3(b) and (c) that the models recognize some of the samples of minority classes. This can also be observed in the improvement of F1-score parameters in the WCCE case. In the case of the EC experiment for five classes, the healthy class is recognized more efficiently than other cases (Fig. 3(d–f)). The recognition of minority class led to better F1-score, precision, and recall parameter values among other experiments for five classes.

The results of the three-class classification on EO and EC samples are shown in Tables 4 and 5, respectively, for window-level and patient-level decisions. The three majority classes’ experiments perform better than the five-class classification. Due to the prevalence of class imbalance, the performance parameters get affected in five-class classification. The transformer model trained with EO samples shows better performance than EC samples. The EO experiment with the CCE loss function achieved $76.56\%$ accuracy compared to $70.31\%$ accuracy achieved by the EC experiment with focal loss ($\gamma$ = 0.5) in patient-level. The advantage of using focal loss can be seen in the case of precision of EO case with value as $91.07\%$.

**Fig. 4:**
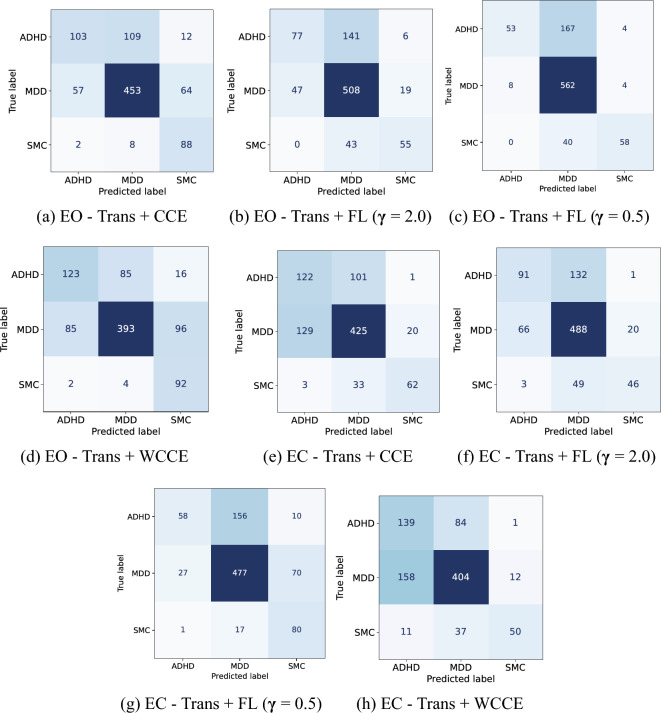
Confusion matrices for the three-class classification eye open and eye closed samples

Figure 4 shows the confusion matrices for the three-class classification for window-level decisions. As shown in Fig. 4(a)  and (d), for the EO experiment using CCE and WCCE loss functions, ADHD and SMC classes are classified better in comparison to MDD class. A similar pattern is observed in the case of EC experiments using CCE and WCCE loss functions (Fig. 4(e) and (h)). In case of MDD class, the EO and EC experiments using focal loss function shows higher number of true positives as shown in Fig. 4(c) and (f).

**Table 4:** Performance of transformer model for EEG signals with eyes open on different methods for three class (ADHD, MDD, and SMC) classification

Decision	Method	Accuracy	F1-score	Precision	Recall
Window-level	Trans + CCE	71.87	**66.57**	65.57	71.56
	Trans + Focal Loss ($\gamma$ = 2)	71.43	62.10	68.09	59.67
	Trans + Focal Loss ($\gamma$ = 0.5)	**75.11**	63.87	**82.62**	60.25
	Trans + WCCE	67.86	64.01	61.73	**72.42**
Patient-level	Trans + CCE	68.75	60.01	60.37	61.87
	Trans + Focal Loss ($\gamma$ = 2)	73.44	63.96	74.84	60.36
	Trans + Focal Loss ($\gamma$ = 0.5)	**76.56**	65.75	**91.07**	60.71
	Trans + WCCE	70.31	**67.04**	64.78	**74.39**

The best performance is in bold

**Table 5:** Performance of transformer model for EEG signals with eyes close on different methods for three class (ADHD, MDD, and SMC) classification

Decision	Method	Accuracy	F1-score	Precision	Recall
Window-level	Trans + CCE	67.97	**64.86**	66.25	**63.92**
	Trans + Focal Loss ($\gamma$ = 2)	**69.87**	60.55	66.21	57.48
	Trans + Focal Loss ($\gamma$ = 0.5)	68.64	59.13	63.61	63.54
	Trans + WCCE	66.18	62.63	**67.15**	61.15
Patient-level	Trans + CCE	67.19	**64.83**	72.75	60.92
	Trans + Focal Loss ($\gamma$ = 2)	70.31	61.19	**75.52**	56.05
	Trans + Focal Loss ($\gamma$ = 0.5)	**70.31**	61.87	66.53	**65.36**
	Trans + WCCE	65.62	60.91	72.62	57.43

The best performance is in bold

**Fig. 5:**
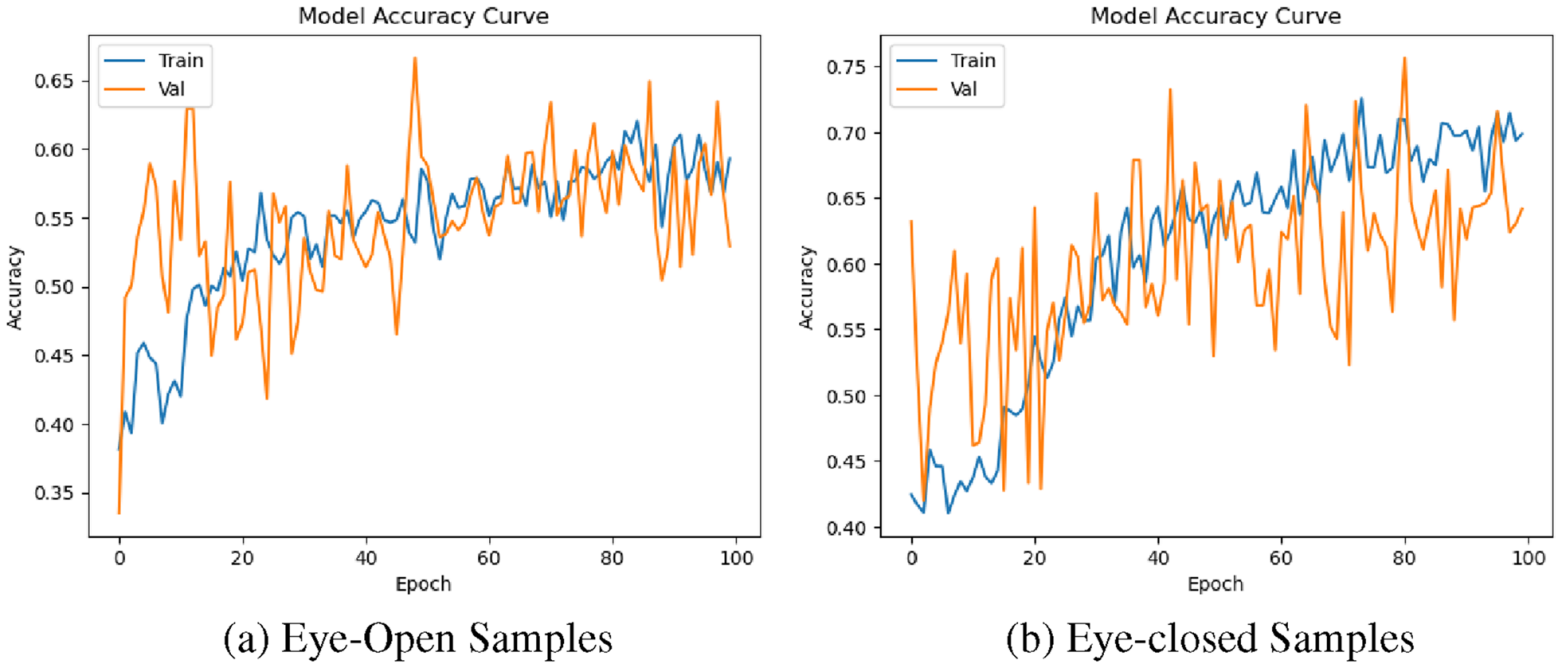
Accuracy plots for five-class classification of eye-open and eye-closed samples (Blue plot: training curve and orange plot: validation curve)

The transformer model was trained for 100 epochs, and the best model was selected using the early stopping and checkpoint method. During training, a checkpoint is created when the validation error is less than the previous epochs, and the current model is saved as the best model. The training and validation accuracy plots for 100 epochs for the five-class classification are shown in Fig. 5. It can be seen from the plot that the best validation accuracy for the eye-open EEG data was identified at epoch number 45 (Fig. 5(a)). Likewise, the best validation accuracy for the eye-closed EEG data was identified at epoch 50 (Fig. 5(b)). Beyond these identified epochs, the model started overfitting the data with no further improvement to validation accuracy.

### Comparision with methods from literature

The proposed transformer model is also compared with other state-of-the-art deep learning-based models that were previously developed for raw EEG-based classification. Two CNN-based methods (i.e. EEGNet [31] and DeepConvNet [32]), originally proposed for classifying raw EEG data for brain–computer interfaces, were selected. The two methods were tested on the TDBRAIN dataset while keeping the same parameters settings according to the original implementations [31, 32]. To ensure an unbiased comparison, our model was compared against the two methods under their optimal original parametrizations for the five-class classification problem. More specifically, the two methods used the CCE loss function for raw EEG classification, therefore, our proposed method with the CCE loss function was used here for comparison purposes (see Table 6). Our proposed method outperformed both methods in terms of Accuracy, F1-score, Precision, and Recall. Interestingly, our proposed transformer-based architecture is relatively close in terms of the number of training parameters (#TP) to the compact EEGNet architecture [31], but have much less parameters than the DeepConvNet method [32], which suggests that our model achieved higher performance with a reasonable number of training parameters.

**Table 6:** Performance comparision of transformer model with state-of-the-art for Raw EEG classification for five classes (ADHD, MDD, OCD, SMC, and Healthy)

Author	Method	Accuracy	F1-score	Precision	Recall	# TP
Eye Open EEG						
Lawhern et al. [31]	EEGNet	54.89	36.53	42.04	41.13	**53.86k**
Schirrmeister et al. [32]	DeepConvNet	59.49	35.77	37.26	37.99	207.53k
Proposed Method	Transformer	**63.21**	**41.99**	**42.51**	**41.49**	72.64k
Eye Close EEG						
Lawhern et al. [31]	EEGNet	55.28	40.84	44.64	42.92	**53.86k**
Schirrmeister et al. [32]	DeepConvNet	59.78	38.04	**55.52**	42.15	207.53k
Proposed Method	Transformer	**61.74**	**46.57**	51.24	**47.83**	72.64k

The best performance is in bold

### Limitations and future work

We observed that the models trained on complex raw EEG data did not provide high performance that could be clinically useful. An attempt has been made to analyze possible limitations and future solutions for classifying the TDBRAIN dataset. TDBRAIN dataset suffers from class imbalance, a classical problem in medical datasets. To address this issue, we have implemented the transformer model with class weights and focal loss function. The results obtained by these methods are not yet suitable for real-life deployment. The transformer model implements a window-level approach based on the assumption that EEG signals of 10 s are robust stationary representations of brain dynamics that are patient-specific.

As future work, we plan to investigate whole EEG signal analysis for mental dysfunction analysis. Various techniques like large margin nearest neighbor, sampling methods for imbalanced classification, and EEG signal augmentation can be implemented to further address the issue of class imbalance. The raw EEG signals are used in this work to train the classification model. Classifying raw EEG signals is challenging, and artifacts present in the signal may hinder the learning process. However, raw EEG signal analysis can open the possibility for near real-time applications for diagnostic purposes, including fast analysis of raw signals from portable EEG devices. Our rationale was also motivated by the fact that different clinical populations might not show similar artifacts (e.g. neurotypicals might display fewer artefacts than people with mental deficits), which could in turn help the classification. Nonetheless, the value of pre-processing should not be overlooked, as it is expected that better performance can be obtained after artefacts removal. Likewise, it is also most likely that spectral features might yield better classification of the different mental conditions than features extracted in the time domain.

## Conclusion

An automated psychiatric dysfunction classification method using raw EEG signals is proposed in this work. The proposed method implements a transformer model with three different loss functions for classification. Two sets of experiments are performed using samples collected during rest from patients with eyes open and closed. The novelty of the work lies in the implementation of transformer models for multi-class classification and the addition of specialized loss functions to address the class imbalance issue in the publicly available TDBRAIN dataset. The approaches are employed for classification into five and three categories of mental dysfunction. The raw EEG signal is taken as input for the transformer block with eight attention heads. The extracted features from the transformer blocks are applied to a fully connected layer and classified using the softmax function. The parametric evaluation of the training and testing phase shows that the transformer with categorical cross entropy shows better accuracy for five-category classification. The transformer with weighted categorical cross entropy performed better in the open-eye case in terms of F1-score, precision, and recall. For the three-category classification case, the transformer with categorical cross-entropy yielded better accuracy for eye-open samples than that with the focal loss for eye-closed samples. Classwise performance of all the models is also analyzed using confusion matrices. The proposed work can be incorporated with real-life clinical systems to analyze and classify neurological and mental dysfunctions. Future work needs to implement robust methods to address the class imbalance in the TDBRAIN dataset.

## Data Availability

The TDBRAIN dataset used for the experimentation of this work is publicly available on Brain Clinics website (https://brainclinics.com/resources/). No new data were created or analyzed in this study. Data sharing is not applicable to this article.
